# Chromatin organization in pluripotent cells: emerging approaches to study and disrupt function

**DOI:** 10.1093/bfgp/elv029

**Published:** 2015-07-23

**Authors:** Clara Lopes Novo, Peter J. Rugg-Gunn

**Keywords:** chromatin interactions, genome folding, nuclear organization, gene regulation, pluripotency, embryonic stem cells

## Abstract

Translating the vast amounts of genomic and epigenomic information accumulated on the linear genome into three-dimensional models of nuclear organization is a current major challenge. In response to this challenge, recent technological innovations based on chromosome conformation capture methods in combination with increasingly powerful functional approaches have revealed exciting insights into key aspects of genome regulation. These findings have led to an emerging model where the genome is folded and compartmentalized into highly conserved topological domains that are further divided into functional subdomains containing physical loops that bring *cis*-regulatory elements to close proximity. Targeted functional experiments, largely based on designable DNA-binding proteins, have begun to define the major architectural proteins required to establish and maintain appropriate genome regulation. Here, we focus on the accessible and well-characterized system of pluripotent cells to review the functional role of chromatin organization in regulating pluripotency, differentiation and reprogramming.

## Introduction

Three-dimensional nuclear structure is established and maintained by complex layers of regulatory information superimposed on the genome. Compartmentalization of functional chromosome domains and their correct spatial distribution within the nucleus are required for several important nuclear functions including transcription, repair and replication [1]. In addition, appropriate folding of chromosomes and the formation of physical interactions between regulatory elements are necessary for appropriate gene regulation [2]. Recent technical advances in studying chromosome organization and folding have provided exciting new insights into genome architecture. Emerging models suggest that nuclear structure is formed by large highly conserved compartments, within which occur dynamic cell type-specific regulatory interactions. A current challenge is to reconcile how these two aspects of nuclear organization are coordinated to control overall genome function.

Pluripotent cells, which consist of embryonic stem (ES) cells and their reprogrammed counterparts, induced pluripotent stem (iPS) cells, are one of the most well-characterized cellular systems to study the connection between chromatin organization and cell identity [3]. Extensive chromatin and transcriptional reorganization occurs on pluripotent cell differentiation and reprogramming, enabling the mechanisms that regulate genome architecture to be examined during these dynamic processes. Profiling of genomic features and epigenomic modifications have revealed an enormous amount of information about the linear genome of pluripotent cells, including detailed descriptions of chromatin states and the location of regulatory elements [4, 5]. An important task is to now develop new technologies that will allow the translation of linear genome-wide information into three-dimensional models of genome organization, and the provision of a new set of functional tools to test these models.

Here, we highlight several emerging technologies that enable the study and functional analysis of chromatin organization, with a particular focus on their application to pluripotent cells. We review how these recent findings have contributed to our understanding of genome function and discuss potential future applications of these novel approaches.

### Microscopy-based techniques to profile nuclear organization

Microscopy-based techniques were the first to reveal large-scale structural organization in the genome, when Emil Heitz observed differences in chromosome condensation patterns within interphasic nuclei [6]. Subsequently, euchromatin and heterochromatin compartments have been well characterized and overall correlate with active and inactive regions of the genome, respectively [7]. Heterochromatic regions tend to be gene poor and are enriched at the nuclear periphery and around the nucleoli, whilst euchromatin is typically localized to the nuclear interior [8]. The non-random physical segregation of heterochromatin and euchromatin is thought to promote the three-dimensional organization of the genome and may have a functional role in gene regulation [9–12].

More recent microscopy-based techniques have advanced our understanding of nuclear organization and how this impacts on cell regulation and function. In particular, an unusual chromatin organization has been observed in mouse ES cells and fully reprogrammed iPS cells, which may be linked to their pluripotent status. For example, visualization of chromatin with DNA-binding dyes or indirectly with antibodies against heterochromatin-binding protein HP1 or heterochromatin-associated histone modifications, such as histone H3 lysine 9 trimethylation (H3K9me3), have shown that mouse ES cells have fewer but larger heterochromatin foci [13, 14]. These findings were validated using alternative assays of chromatin organization, including DNA fluorescence *in situ* hybridization (FISH) against major satellite repeats that occur within pericentromeric heterochromatin [13]. Furthermore, electron spectroscopic imaging, a direct and quantitative readout of chromatin fibre organization, revealed that chromatin is uniformly dispersed in mouse pluripotent cells, with little compacted chromatin [14, 15]. Excitingly, this form of nuclear architecture appears to be conserved in pluripotent human cells, particularly those maintained in the newly described ‘naïve’ state [16].

**Figure 1.**
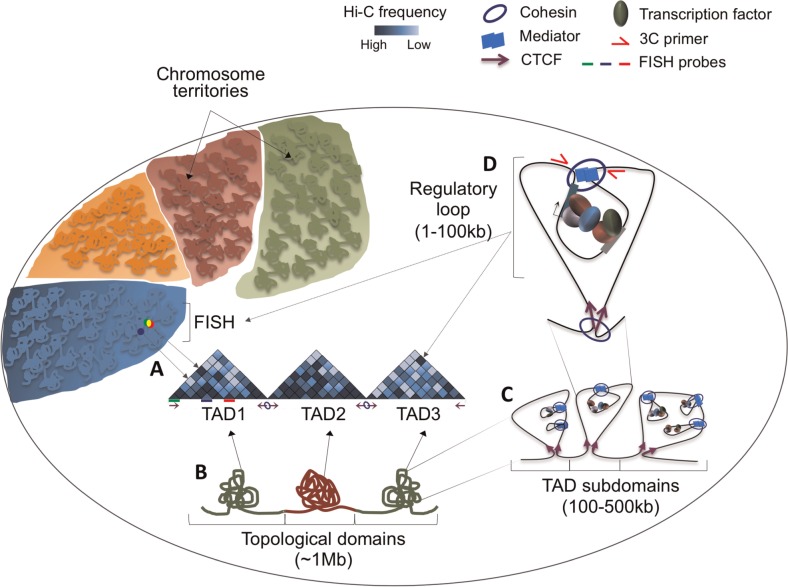
Several layers of genome folding ensure higher order genome organization. Genomes are folded and distributed into CT. Within each CT, further folding delimited by converging CTCF binding sites (purple arrows) form ∼1 Mb topological domains (TADs). (**A**) FISH probes label three loci within TAD1. The close overlap between green and red FISH probes provides validation for a strong intra-TAD interaction between the genomic loci represented by green and red lines underneath the Hi-C heatmap. The high-frequency interaction is indicated as a dark blue bin in the heatmap. The blue FISH probe illustrates a locus that rarely interacts with the green locus within a folded chromosome, despite being in close linear proximity. (**B**) TADs associate with either open [26] or repressed [27] chromatin states. A specific locus intermingles more frequently within the same TAD, as represented in the Hi-C interaction heatmaps (darker blue: higher frequency of interactions; lighter blue: lower frequency of interaction). (**C**) Each TAD is further organized into subdomains that (**D**) are then folded in regulatory loops (dark blue circle in TAD3), where *cis*-regulatory regions are bound by transcription factors and contact promoters to regulate its function. Subdomains and regulatory loops are established and maintained by cohesin, mediator and converging CTCF. Interactions can be validated by FISH or by 3C. (A colour version of this figure is available online at: http://bfg.oxfordjournals.org)

FISH studies have further revealed sub-megabase changes in chromatin condensation at specific loci that occur on *in vitro* and *in vivo* cell differentiation [17–19]. Functionally, FISH has also been essential to test the role of tissue-specific *cis*-regulatory elements by probing the colocalization of long-range enhancers and their target genes [20–22]. FISH has revealed that certain genes (*HoxB* or *uPA*) can loop out of their chromosome territories (CT) on gene activation [17, 23]. Interestingly, FISH experiments showed that pluripotency genes tend to relocate from the nuclear interior towards the nuclear periphery on ES cell differentiation, a phenotype associated to changes in gene expression [24]. Despite the tremendous progress in understanding chromatin conformation using FISH and live-cell imaging, the constraint of profiling a limited number of loci simultaneously and the limited spatial resolution has so far prevented the application of FISH for genome-wide profiling [25] (Figure 1A).

### Chromosome conformation capture techniques define the structural domains of genome organization

Development of chromosome conformation capture, commonly referred as ‘3C technology’ [28, 29], has enabled the study of chromosome organization in a genome-wide manner. In 3C approaches, chromatin is cross-linked with formaldehyde, digested with a restriction endonuclease and the loci that are in close-spatial proximity are ligated together, thereby producing hybrid molecules. The resulting hybrid molecules are identified by polymerase chain reaction or high-throughput sequencing, and the frequency of hybrid molecules can be calculated to infer the frequency at which two distinct loci are in close spatial proximity in a cell population. Variations of the original 3C protocol include circular 3C or 3C-on-chip (4C) [30–32]; 3C carbon copy (5C) [26] and 3C followed by high-throughput sequencing (Hi-C) [33] and facilitate the simultaneous survey of multiple interacting loci without requiring any *a priori* knowledge. Other adaptations to the technology include ChIA-PET, which bridges the characterization of an interactome with functional studies by using an antibody-based enrichment step for interactions that occur in the presence of a protein of interest [34].

Information acquired by 3C technologies has advanced the understanding of chromosome folding to unprecedented levels. Within CT [35–37], interphase chromosomes are folded into megabase-sized domains and smaller sub-domains known as topologically associating domains (TADs) [38–42]. Functional analysis of TADs revealed the presence of ‘active’ and ‘repressed’ TAD domains, thereby reconciling higher-order chromatin organization with what was previously known as ‘A’ and ‘B’ compartments [33, 38] (Figure 1B). These compartments are associated with functional partitions of chromatin, including differences in DNA replication timing and the presence of particular chromatin modifications [43]. Because TADs are defined by the frequency and directionality of interactions, gene regulatory interactions between enhancers and promoters are more likely to occur within the same compartment or domain, suggesting that TADs might be intrinsically functional domains [44]. Furthermore, several studies have shown that the size (∼1 Mb) and boundary location of TADs are largely invariant between different tissues and species, and thus, TADs could be conserved structural units of chromosome organization [38, 39, 45]. Several FISH studies reassuringly confirmed that loci within the same TAD tend to intermingle more than those in adjacent TADs [39] (Figure 1A). Interestingly, a recent study in human cells revealed that despite conservation of TAD boundaries, 36% of genome compartments switch directionality (active to repressed, or vice versa) on ES cell differentiation [46]. Remarkably, this spatial plasticity is associated with an increase in the proportion of repressed compartments, supporting previous data, suggesting an increase in repressive heterochromatin levels occurs on ES cell differentiation [46–48].

Improvement of 3C-technology resolution has further identified DNA loops connecting *cis*-regulatory elements to promoters, thereby forming subdomains within TADs that can range up to 100 kb in size [44, 49] (Figure 1C). A further exciting development is the ability to use sequence-specific capture approaches to enrich for regions of interest within 3C or Hi-C interaction libraries, a step that overcomes some of the resolution limitations of interaction libraries owing to their high complexity [50–53]. Capture Hi-C has been used to good effect to interrogate the interactome of >22 000 promoters in mouse ES cells, thereby assigning regulatory elements such as enhancers, silencers and boundary elements to their target promoters [53]. These studies are beginning to reveal the general principles of how chromosome folding and DNA loops impact on gene regulation and genome function.

### The role of architectural proteins and master regulators in defining chromosome organization

Cohesin, mediator and CCCTC-binding factor (CTCF) are known architectural proteins that promote and stabilize chromatin looping between distal regulatory elements, including enhancers and promoters [54, 55]. The development of 3C technology has greatly facilitated the study of architectural and other structural proteins in chromatin organization. For example, depletion of mediator or cohesin from mouse ES cells disrupts self-renewal and induces cell differentiation [54, 56]. ES cell super-enhancers, a subgroup of enhancers highly enriched for architectural proteins, histone modifications such as H3K27ac and H3K4me1 and master transcription factors [57] are particularly sensitive to loss of mediator [54]. ChIA-PET of CTCF in ES cells identified CTCF-mediated chromatin loops and revealed the existence of CTCF delineated chromatin compartments with distinct transcriptional and epigenetic states and lamin-associated silenced domains [58]. Thus, it has been proposed that CTCF, cohesin and mediator facilitate functional regulatory interactions by promoting spatial clustering while ‘insulating’ inappropriate promoter-enhancer looping [45, 54, 59] and that chromatin loop domains are anchored by these architectural proteins [42, 45, 59–61]. Interestingly, high-resolution Hi-C revealed that two converging CTCF binding sites are required to anchor the looping of regulatory domains, revealing a previously unappreciated requirement for protein binding orientation in chromosome folding [41]. Surprisingly, depletion of cohesin in differentiated cells (thymocytes) does not seem to impact enhancer or super-enhancer function, suggesting that the topological organization of ES cells might be more tightly linked to a regulatory function, as compared with differentiated cells [62].

Key pluripotent transcription factors have also been implicated in chromatin looping in ES and iPS cells. Depletion in ES cells of transcription factors OCT4, KLF4 or SOX2 leads to the transcriptional down-regulation of genes associated with super-enhancers, disturbs enhancer–promoter loops and promotes differentiation [57, 63–65]. Furthermore, pluripotency-specific interactomes have been described around the *Nanog* and *Oct4* promoters [64, 66], and differentiation events were shown to disrupt promoter–enhancer looping at these loci [42, 54, 67, 68]. Excitingly, intertwining of pluripotency-transcription factors might be required to establish the pluripotent interactome. For example, OCT4 is required to maintain appropriate chromatin structure at the *Nanog* locus [69].

Chromatin loops are also associated with changes in chromatin organization that occur on cell differentiation and reprogramming. For example, differentiated cells have distinct interactomes at several key loci when compared with their originating ES cells [42], suggesting that chromatin reorganization occurs during differentiation at least at the level of regulatory loci. Additionally, a pluripotency-specific interactome anchored around the *Nanog*-promoter is re-established during iPS cell reprogramming, and this is dependent on transcription factor and architectural protein occupancy [66]. During reprogramming of human cells, binding of ectopic OCT4 and NANOG to specific loci can be similar between iPS cells and non-reprogrammed cells, but in contrast, enhancer–promoter looping at the *OCT4*, *SOX2* and *NANOG* loci is specific to iPS cells and correlated with complete reprogramming and active transcription of these endogenous loci [70]. Furthermore, despite the equal loading of the key transcription factors in iPS cells and non-reprogrammed cells, the pluripotent-specific interactomes are only established in the presence of cohesin and mediator [64, 70]. These findings uncovered a novel epigenetic barrier to pluripotency: recruitment of cohesin and mediator to establish the pluripotent-specific permissive chromatin interactions that, in some cases, might be facilitated by protein–protein interactions with transcription factors [64].

Supporting a model where cell-identity-specific transcription factors play a dominant role in chromatin organization, a study applied ChIA-PET and chromatin immunoprecipitation to identify RNA polymerase II-mediated chromatin interactions in ES cells and B-cells and found a correlation for cell-specific transcription factors in the establishment of chromatin structure [71]. Astoundingly, 65% of the enhancers analysed were shown to undertake long-range interactions that jumped over several non-interacting gene loci [71]. Together, these studies elaborate a model in which architectural proteins and cell-type-specific master regulators act together to partition the genome into functional domains (Figure 1D). On cell differentiation, changes in the occupancy of architectural proteins and, in particular, of master regulators are associated with the formation of new functional units and frequently a change in chromatin and transcriptional state of existing domains. Further defining the extent to which these events contribute to cell identity is currently an active area of research, and will depend, in part, on the development of new tools to perturb genome organization.

## Functional analysis of genome organization

Experiments aimed at determining the causative role of genome organization in controlling nuclear function and gene regulation have been challenging because many of the available approaches result in genome-wide perturbation, and can therefore lead to difficulties in distinguishing between cause and consequence. Nevertheless, as described above, many landmark studies have successfully used gain and loss of function studies to define the major factors required for genome organization in stem cells and development including architectural proteins, chromatin regulators and pluripotency transcription factors [72–74]. Although insightful, genome-wide perturbation may not be suitable for all functional studies. More focused approaches that tether specific factors to target sites in the genome have revealed important insights into transcription factor and coactivator recruitment [75]. Many of these methods, however, suffer the requirement of transgene integration, often in large copy number, which perturb the genomic sequence and context. Building on this body of work, a new toolkit of designer DNA-binding proteins now enable the targeted investigation of specific sites within the genome, allowing more precise and controlled perturbations without transgene integration.

The new classes of DNA-binding factors include proteins within the zinc-finger family, transcription activator-like effectors (TALE) and clustered regularly interspaced short palindromic repeats (CRISPR). All three protein classes have important and diverse biological roles, but their uptake as sequence-specific genome engineering tools has been driven by rapid technological developments that have repurposed their biological function [76–82]. DNA-binding proteins are constructed from predictable or modular components and can therefore be designed to bind to any target sequence. In addition, the proteins are customizable and versatile, thereby enabling their fusion with a wide range of effector domains that expand their utility. Here, we focus on several of these new approaches and present examples that highlight how they can be used to study genome organization at all levels.

## Visualization of nuclear domains

**Figure 2.**
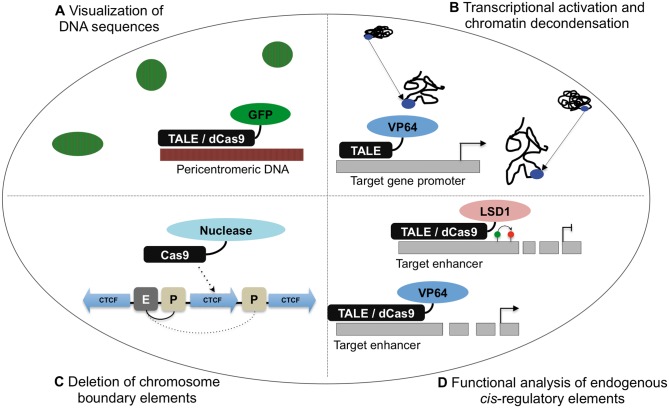
Application of designer DNA-binding proteins to examine genome function. (**A**) TALE-GFP and dCas9-GFP fusion proteins can be targeted to DNA sequences, enabling the visualization of genome compartments in live cells. Example shown is for pericentromeric heterochromatin DNA, which cluster to form chromocentres in mouse cells. (**B**) The strong VP64 transactivator domain can be fused to TALE proteins, leading to transcriptional up-regulation of target genes. Example shown illustrates the repositioning within the nucleus of targeted gene regions and local chromatin decondensation that are caused by transcriptional up-regulation, as revealed by DNA FISH. (**C**) Targeting of Cas9-nuclease proteins to delete chromosome boundary elements. Example shown illustrates deletion of a CTCF binding site, which can result in the expansion of localized gene loops into neighbouring regions. E, enhancer; P, promoter. (**D**) TALE and Cas9 proteins can enable the investigation of *cis*-regulatory elements in several ways. Upper schematic represents TALE-LSD1 decommissioning of an enhancer element by demethylating the associated histone proteins, which can result in the transcriptional down-regulation of a nearby gene(s). Lower schematic shows targeting of TALE-VP64 or dCas9-VP64 to an enhancer element, which can result in the transcriptional up-regulation of a nearby gene(s). (A colour version of this figure is available online at: http://bfg.oxfordjournals.org)

A recent methodological advance is the design of systems that enable live imaging of nuclear organization and sub-nuclear compartments in individual cells, which is attractive for studying dynamic processes such as the cell cycle and chromatin structure. Endogenous repetitive genomic sequences, including centromeres, telomeres and pericentric heterochromatin, can be visualized through sequence-specific TALEs fused to monomeric green fluorescent protein (GFP) (Figure 2A) [83, 84]. When expressed in mouse ES cells, the majority of the GFP signal showed strong enrichment in the target genome compartment. Importantly, the TALE-GFP did not alter the nuclear organization of target regions, demonstrating the ability to visualize DNA sequences with minimal perturbation. In a further powerful example of the specificity of the technology, the TALE-GFP could be designed over sequences containing single nucleotide mismatches between alleles, enabling allele-specific visualization of repetitive sequences [83]. This is a major advance for the study of genome organization in the very early stages of mammalian development, when parental genomes are remodelled [85].

A related approach using an optimized CRISPR/Cas system to visualize specific DNA loci in live cells was recently described [86, 87]. Here, GFP was fused with a nuclease-deficient Cas9 (dCas9). When co-expressed with an optimized guide RNA targeting telomere sequences, dCas9-GFP was detected in sharp puncta that overlapped with telomere DNA FISH signals [86]. The system was used to good effect to monitor telomere dynamics in live cells using time-lapse microscopy. In addition, non-repetitive genomic sequences could be visualized when targeted by an array of 36 guide RNAs that tiled a 2 kb region, demonstrating the broader applicability of this technology [86].

The TALE and Cas9 approaches open up an exciting new area of cell biology, enabling real-time experiments to track the movement of chromatin and specific DNA regions, as well as imaging through cell division and other cell processes. The technology is remarkably flexible and customizable, ensuring their wide adoption and use in the field. Several technical challenges remain. One is that the targeting of the TALE and Cas9 proteins must be carefully designed to minimize the likelihood of off-target effects caused by transcriptional changes and/or blocking access of other DNA-binding elements to their regulatory sequences. A second challenge is that some background signal was detected in the TALE and Cas9 studies, presumably owing to unbound TALE-GFP or dCas9-GFP proteins. Further technical developments are therefore required to improve the signal to noise ratio, and this should also lead to improvements in the ability to visualize non-repetitive sequences.

## Chromatin condensation and positioning

As described earlier, the position of gene loci within the nucleus is correlated with transcriptional regulation, with localization at the nuclear periphery associated with transcriptional down-regulation [27, 88–90]. Several new tools have been developed that enable the exploration of mechanisms that connect the transcriptional status of a particular DNA sequence with nuclear position. In one recent study, three genes that are transcriptionally up-regulated on mouse ES cell to neural progenitor cell differentiation were used as a model system because they show consistent repositioning from the nuclear periphery to the nuclear interior during differentiation [91]. TALE-VP64 fusion proteins targeted to the promoters of each of the three gene loci triggered robust transcriptional up-regulation and localized chromatin decondensation. Excitingly, these events were sufficient to cause the relocalization of the targeted loci towards the nuclear interior in ES cells (Figure 2B). In a further experiment, a TALE fusion protein was generated with a short acidic domain that is known to decondense chromatin without change in gene transcription. On expression of these proteins, chromatin decondensation was sufficient to trigger nuclear repositioning of the targeted loci from the periphery to the interior, leading to the conclusion that transcriptional up-regulation was not a requirement for this process. These novel approaches demonstrate the potential for targeting particular chromatin modulators to specific sites in the genome.

## Functional analysis of gene loops and chromosome boundary element

Gene loops can be identified from genome-wide DNA proximity ligation assays and are often anchored at either end by architectural components such as mediator, cohesin and CTCF [42, 59–61]. Until recently, it was unclear whether the anchors were required to maintain the gene loops and regulate genes within each loop. To address this, CRISPR/Cas technology was used to delete CTCF binding sites at the boundaries of five gene loops in mouse ES cells, thereby removing the anchor at one end of each loop [92]. Impressively, deletion of a CTCF binding site led to altered expression of genes immediately outside of the gene loop in all five targeted regions (genes were transcriptionally up-regulated in four of five regions, and down-regulated in one of the regions) (Figure 2C). Furthermore, deletion of CTCF sites was often associated with transcriptional changes of genes inside the gene loop (for three of the five regions). Another interesting result from this series of experiments is the long-range effect of some of the deletions. For example, a gene 100 kb away from the deleted CTCF site was transcriptionally up-regulated. 3C analysis for some of the loci revealed that genes newly activated on CTCF binding-site deletion formed new DNA interactions with a nearby *cis*-regulatory region, in line with the conclusion that the CTCF sites can form boundaries that insulate genes in one neighbourhood from another [92]. A recent study used a similar strategy to delete CTCF sites within *Hox* clusters in mouse ES cells [93]. Excitingly, loss of CTCF binding resulted in the spreading of active chromatin into repressive chromatin domains, leading to ectopic activation of several *Hox* genes on cell differentiation. These results reveal that CTCF helps establish functional domains that control developmental gene regulation. Our understanding of chromosome boundaries is still far from complete, however, as it is not possible to predict which genes will be transcriptionally altered on loss of boundary and other structural elements. Nevertheless, the combination of DNA interaction methods and functional analysis of target regions will ensure exciting progress towards these goals.

## Perturbation of *cis*-regulatory element function

Identification of *cis*-regulatory elements using chromatin signatures, 3C and functional approaches is now well established [94]. One emerging challenge is how to connect each individual regulatory unit with their associated gene promoters in a given cell type, and determine the functional consequences of disrupting enhancer elements on gene regulation.

Towards this goal, several recent publications have devised new technical approaches to the functional analysis of *cis*-regulatory elements (Figure 2D). In one insightful set of experiments, artificial zinc-fingers were used to tether specific looping factors to the *β-globin* promoter in erythroid cells [95]. This led to the formation of new interactions with the locus control region and transcriptional up-regulation of *β-globin*, thereby providing persuasive evidence that chromatin looping has a causative role in gene regulation. Similarly, alternative DNA-binding proteins such as TALE-VP64 and dCas9-VP64 can be targeted to *cis*-regulatory elements and exert a strong transcriptional response of nearby genes [96–98]. This approach has the advantage in functional studies of modulating endogenous gene levels, rather than relying on transgene overexpression. A direct comparison between TALE-VP64 and dCas9-VP64 revealed that both systems could activate reporter plasmids and endogenous transcription; however, TALE-VP64 proteins were more effective in eliciting a functional change [99]. In this example, *Oct4* and *Nanog cis*-regulatory elements were targeted, and only TALE-VP64 up-regulated *Nanog* levels sufficiently to enable reprogramming to pluripotency. One important consideration is that dCas9-VP64 prevented the binding of other native transcription factors that co-bind the *Oct4* and *Nanog* enhancers, and the authors suggested these unexpected effects were partially responsible for the inability of dCas9-VP64 to reprogramme cells [99].

A further example of this approach was used to target TALE-VP64 to specific *cis*-regulatory elements in human K562 cells [100]. A robust transcriptional increase (3–22 fold) of the gene predicted to be regulated by the enhancer was detected, with little effect on other genes in the vicinity. Associated epigenetic marks of active enhancers were also elevated. It is unclear how TALE-VP64 mediates this effect, but it is likely that VP64 recruits chromatin remodellers and co-activators, resulting in the acquisition of an epigenetic state that is characteristic of active regulatory sequences [101]. One potential use of this technology would be to ask how frequently does a newly activated *cis*-regulatory element interact with the predicted gene promoter, and how might this be regulated? In this regard, the cell context is clearly important here, because activation of the same *cis*-regulatory elements in alternative cell types did not cause transcriptional changes. Whether these differences are determined by available cofactors or the epigenetic state of the *cis*-regulatory elements is not clear. Nevertheless, when used in the appropriate context, this technology can be effective. The authors of this study, for example, showed that activation of enhancer elements that control blood lineage regulators during mouse ES cell differentiation could dramatically alter the efficiency of haematopoietic differentiation [100].

Several studies have perturbed the function of *cis*-regulatory elements using TALE and Cas9 technologies [102, 103]. In one study, the authors used 3C, bioinformatic and reporter assays to identify regulatory elements that interacted with the *Sox2* locus in mouse ES cells [103]. Focusing on an element ∼100 kb downstream from the *Sox2* transcriptional start site, the authors used CRISPR/Cas9 to delete a 7.3 kb target region that contained several DNA sequences with enhancer activity. Importantly, as the authors anticipated that deletion of the *Sox2* enhancer from both alleles would be lethal to the ES cells, they chose to use a hybrid mouse ES cell line, thereby allowing allele-specific deletion and gene expression analysis. On deletion, there was an 8-fold reduction in *Sox2* transcripts from the same allele, demonstrating that this novel *cis*-regulatory element contributes to the transcriptional regulation of a key pluripotency factor in ES cells. Interestingly, there was a transcriptional increase of the non-targeted allele, suggesting the presence of feedback mechanisms compensating for loss of *Sox2* expression [103]. The combination of enhancer identification followed up by targeted functional analysis provides a valuable framework for future studies examining gene–enhancer interactions. Further refinements to the methodology could include the ability to replace interacting regions with mutated versions, to better define the role of DNA-binding factors in gene-enhancer looping.

In addition to enhancer deletions, another elegant way to test the function of *cis*-regulatory elements is to use epigenomic engineering methods to inactivate, or decommission, active enhancers in their native context. This approach was recently achieved using TALE-LSD1 fusion proteins, which are predicted to demethylate H3K4 histone proteins at active enhancers, thereby perturbing the function of the enhancer [104]. The authors targeted 40 *cis*-regulatory elements, and identified 26 (65%) with the predicted decrease in histone modifications associated with active enhancers. For several targets (but not all), transcription of a nearby gene was also altered, suggesting that regulatory function was perturbed. This is an exciting first step in using epigenomic engineering to alter *cis*-regulatory element function, and further refinements to the technology, such as combinatorial approaches, will provide a powerful platform to push this work forward.

## Perspectives

The combination of microscopy, 3C technologies and functional approaches has tremendously advanced our knowledge of genome organization. Identification of the general principles that govern chromatin compartmentalization and physical interactions between regulatory elements have led to a better understanding of cell identity and potentially mechanisms to control cell state changes during pluripotent cell differentiation and reprogramming.

Despite the exciting progress in this area, several methodological challenges remain to be overcome. First, because interaction data sets are created from static populations of cells, they represent only the topological tendency of the population. Nagano and colleagues have provided the first attempt to bypass this population-biased limitation by developing a single-cell Hi-C technique [105], and further refinements to this remarkable technology will yield important insights into considerations such as cell heterogeneity. Second, Hi-C and ChIA-PET data sets contain only a minor proportion of all interactions owing to limitations in sequencing depth and coverage [55]. These limitations are starting to be overcome by new capture methods that enrich Hi-C-hybrid products of interest before sequencing, allowing improved coverage and resolution [50–53] or by mapping global chromatin interactions on the basis of random fragmentation with DNase I (DNase Hi-C) [106]. Third, a major limitation is that static interactomes do not consider the dynamism of chromatin through cellular process, including cell division or signalling responses. To transit from a 3D to 4D understanding of nuclear architecture, epigenome profiling must incorporate spatiotemporal alterations. Fourth, formaldehyde-based cross-linking studies must consider the propensity of artefact nuclear aggregates [107, 108]. Indeed, these artefacts may give rise to false positive interactions, especially between decondensed loci as elegantly demonstrated in one recent study [109]. Therefore, interpretation of interactions identified by 3C technology should ideally be validated with a technique that does not involve cross-linking, such as FISH. Fifth, the vast amount of sequencing data that are being generated requires incredible computational power and intensive analysis, which are not readily accessible to all researchers. Standardized bioinformatic pipelines should be established to facilitate the comparison of findings from different laboratories. Sixth, although designable DNA-binding proteins offer a powerful and flexible approach to study the functional aspects of chromatin organization, care must be taken to control for off-target effects and the potential for altered or impaired binding of nearby native transcription factors.

In summary, methods to study and perturb genome organization are enabling exciting new insights into nuclear function. Progress in this area will better define how chromosomes are compartmentalized into functional domains, and how physical interactions between regulatory sequences are formed and regulated, which together will further our understanding of the relationship between three-dimensional organization and genome function.

Key Points

- Three-dimensional nuclear organization contributes to genome folding, chromosome compartmentalization and the formation of gene regulatory interactions, ensuring appropriate genome function.
- Recent technological innovations including chromosome conformation capture approaches have revealed the presence of major structural and functional domains within the genome.
- Architectural proteins and master transcription factors coordinate aspects of genome organization to ensure transcriptional states and appropriate cell identity.
- Designable DNA-binding proteins have been repurposed to enable the visualization and targeted perturbation of genome organization, thereby providing new functional insights.
